# Molecular diagnosis of citrin deficiency in an infant with intrahepatic cholestasis: identification of a 21.7kb gross deletion that completely silences the transcriptional and translational expression of the affected *SLC25A13* allele

**DOI:** 10.18632/oncotarget.19901

**Published:** 2017-08-03

**Authors:** Zhan-Hui Zhang, Wei-Xia Lin, Qi-Qi Zheng, Li Guo, Yuan-Zong Song

**Affiliations:** ^1^ Clinical Medicine Research Institute, The First Affiliated Hospital, Jinan University, Guangzhou 510630, China; ^2^ Department of Pediatrics, The First Affiliated Hospital, Jinan University, Guangzhou 510630, China

**Keywords:** citrin deficiency, intrahepatic cholestasis, *SLC25A13*, mutation, Western blotting

## Abstract

Neonatal Intrahepatic Cholestasis caused by Citrin Deficiency (NICCD) arises from biallelic *SLC25A13* mutations, and *SLC25A13* analysis provides reliable evidences for NICCD definite diagnosis. However, novel large insertions/deletions in this gene could not be detected just by conventional DNA analysis. This study aimed to explore definite diagnostic evidences for an infant highly-suspected to have NICCD. Prevalent mutation screening and Sanger sequencing of *SLC25A1*3 gene just revealed a paternally-inherited mutation c.851_854del4. Nevertheless, neither citrin protein nor *SLC25A13* transcripts of maternal origin could be detected on Western blotting and cDNA cloning analysis, respectively. On this basis, the hidden maternal mutation was precisely positioned using SNP analysis and semi-quantitative PCR, and finally identified as a novel large deletion c.-3251_c.15+18443del21709bp, which involved the *SLC25A13* promoter region and the entire exon 1 where locates the translation initiation codon. Hence, NICCD was definitely diagnosed in the infant. To the best of our knowledge, the novel gross deletion, which silenced the transcriptional and translational expression of the affected *SLC25A13* allele, is the hitherto largest deletion in *SLC25A13* mutation spectrum. The Western blotting approach using mitochondrial protein extracted from expanded peripheral blood lymphocytes, of particular note, might be a new minimally-invasive and more-feasible molecular tool for NICCD diagnosis.

## INTRODUCTION

The human gene *SLC25A13* is localized at chromosome 7q21.3, which consists of 18 exons and encodes a 3.4kb transcript with a predicted opening reading frame (ORF) of 2,025 bp [1]. The protein product of this gene is designated as citrin, which, as the liver-type aspartate/glutamate carrier isoform 2 (AGC2) [2, 3], functions to export aspartate from the mitochondrial matrix in exchange for cytosolic glutamate and H^+^, playing important roles in the urea cycle and malate-aspartate shuttle [2–6]. Biallelic *SLC25A13* mutations result in citrin deficiency (CD), a disease entity with three age-dependent clinical phenotypes, i.e. Neonatal Intrahepatic Cholestasis caused by Citrin Deficiency (NICCD, OMIM#605814) in neonates or infants [7–9], adult-onset citrullinemia type II (CTLN2, OMIM#603471) in adolescents/adults [1], and Failure to Thrive and Dyslipidemia caused by Citrin Deficiency (FTTDCD) between NICCD and CTLN2 stages [10–14]. As a worldwide distributed disease entity, CD is relatively more common among East Asian population [11, 13–25] while some cases in western countries have been reported [26–32].

NICCD typically exhibits prolonged jaundice, growth retardation, fatty liver, hypoglycemia, hypoproteinemia as well as multiple aminoacidemia [13]. Nevertheless, as of today, there are no well-recognized clinical and biochemical criteria for NICCD diagnosis, and *SLC25A13* gene and/or its expression product analysis has been taken as the reliable diagnostic tools. To date, a total of 106 pathogenic *SLC25A13* mutations/variations have been reported [11, 14, 22, 25, 32–41], most of which are point mutations or short insertions/deletions. Among the *SLC25A13* mutations detected in Chinese CD patients, c.851_854del, c.1638_1660dup, c.615+5G>A and IVS16ins3kb account for about 85% of all mutated alleles [11, 25, 36]. Nowadays, these four high-frequency mutations have been screened by conventional means of Polymerase Chain Reaction (PCR), Long and Accurate PCR (LA-PCR) and PCR-Restriction Fragment Length Polymorphism (PCR-RFLP) procedures, respectively, as the basic molecular targets for NICCD diagnosis. If only one or none such mutation was identified in patients highly suspected to have NICCD, Sanger sequencing of all the *SLC25A13* exons and their flanking sequences would be performed to identify the possible novel mutation [11, 25, 33–35].

The above conventional DNA analytic methods, however, are not applicable for detecting novel large *SLC25A13* insertion/deletion mutations, and the identification of such gross mutation remains a big challenge for the in-time diagnosis of NICCD subjects. In such cases, *SLC25A13* cDNA cloning analysis with human peripheral blood lymphocytes (PBLs) has been developed as a less invasive and more feasible diagnostic tools [11, 25, 34, 35]. Regarding citrin protein detection, Western blotting analysis using biopsied liver specimens or cultured skin fibroblasts has proven to be effective [1, 19, 26, 27, 42], but obtaining these samples is invasive and hence not always feasible in clinical practice. Fresh human PBLs had been tried as the protein source to diagnose CD [43]; however, although not infeasible, this approach is a little bit difficult to repeat, not only in our group, but also in others [42]. This is actually not surprising since the amount of mitochondria as well as the quantity of citrin protein in PBLs is rather limited due to the rather limited cytoplasm in contrast with the large nucleus size in such cells. Moreover, it is not always possible to obtain enough human lymphocytes for investigation, especially from little infants with NICCD.

In this study, a Western blotting approach was developed using the mitochondrial protein extracted from expanded PBLs, and citrin signal was well detected in the healthy control while absent in NICCD patients. On this basis, a novel large *SLC25A13* deletion was precisely positioned and identified via sophisticated molecular analyses, including cDNA cloning, semi-quantitative PCR, family single nucleotide polymorphism (SNP) analysis and LA-PCR. We herein reported the clinical and molecular findings.

## RESULTS

### Clinical findings

A 3-month-old male infant was referred to our hospital due to prolonged jaundice lasting for about 3 months. His jaundiced skin appeared shortly after birth. At the age of 1.2 months, a liver function test was abnormal in the local hospital, and hence breastfeeding was stopped while lactose-restricted formula was introduced to evaluate the possibility of galactosemia. Since jaundice and liver dysfunction did not improve, the infant was referred to another hospital at 1.9 months of age, where increased serum aspartate aminotransferase (AST), gamma-glutamyl transpeptidase (GGT), alkaline phosphatase (ALP), total bilirubin (TBil), direct bilirubin (DBil), indirect bilirubin (IBil) and total bile acids (TBA) as well as decreased total protein (TP), albumin (ALB) and globulin (GLB) were unveiled (Table 1). Subsequent tandem mass spectrometry (MS-MS) analysis detected elevated citrulline, threonine, tyrosine and methionine in dried blood samples, while large quantities of 4-hydroxyphenyllactic and 4-hydroxyphenylpyruvic acids were detected on urinary gas chromatography-mass spectrometry (GC-MS) analysis. The infant was thus highly suspected to have NICCD, and was referred to our hospital for *SLC25A13* analysis at the age of 3 months. As the first baby of a non-consanguineous couple, the patient was born at term with the birth weight of 3.1kg and body length of 50cm. There was no family history of any genetic disease.

**Table 1:** Biochemical alterations over time in the patient with NICCD

Biochemical indices	Reference ranges	1.9M	2.2M	2.4M	3.0M^a^	4.2M	7.2M	10.4M	15.7M	21.9M
**ALT**	5-40 U/L	35.9	26	24	81	33	52	58	31	22
**AST**	5-40 U/L	191.7	66	74	83	59	60	64	37	37
**GGT**	8-50 U/L	–	209	144	145	158	34	28	11	13
**ALP**	20-500 U/L	1253	806	484	526	514	271	251	370	282
**TP**	60-83 g/L	48.5	50.4	42.7	51.1	58.1	59.6	66.1	64.2	65.9
**Alb**	35-55 g/L	29.3	31.6	26.8	35.8	40.1	43.3	47.5	46.1	48.2
**Glb**	20-30 g/L	19.2	18.8	15.9	15.3	18	16.3	18.6	18.1	17.7
**Tbil**	2-19 μmol/L	273	145.8	116.1	81.1	39.9	5.1	5.6	5.5	9
**Dbil**	0-6 μmol/L	134.4	104.2	79.5	59.4	32	1.2	2.1	2.3	3.1
**Ibil**	3-21 μmol/L	138.6	41.6	36.6	21.7	7.9	3.9	3.5	3.2	5.9
**TBA**	0-10 μmol/L	–	488.4	344.3	169.5	138.8	11.8	10.2	11.8	8.5
**AFP**	0-12 ng/ml	–	–	–	207130	4369.8	–	7.6	–	2.9

M, months (age); ^a^When a lactose-free and MCT-enriched therapeutic formula was introduced; ALT, alanine aminotransferase; AST, aspartate aminotransferase; GGT, γ-glutamyl transpeptidase; ALP, alkaline phosphatase; TP, total protein; Alb, albumin; Glb, globulin; Tbil, total bilirubin; Dbil, direct bilirubin; Ibil, indirect bilirubin; TBA, total bile acids; AFP, alpha-feto protein; -, not tested.

Physical examination at admission revealed a body weight of 4kg (−3.77SD), body length of 56cm (−3.04SD), and head circumference of 39cm (−1.5SD), along with mild jaundiced skin and sclera. The lungs were clear on auscultation, and no abnormal cardiac sounds or murmurs were heard. In addition, no abdominal distention was observed, and no liver or spleen was palpable under the bilateral subcostal margin. On serum biochemistry analysis, the elevated levels of GGT, DBil, TBA and the DBil/TBil ratio indicated intrahepatic cholestasis (Table 1). Notably, the level of alpha-fetal protein (AFP) was up to 207,130 ng/ml.

The diagnosis of NICCD was developed clinically, and he was switched to a lactose-free and medium-chain triglyceride (MCT)-enriched formula at the age of 3 months. His physical and biochemical conditions were improved gradually, and completely resolved at the age of 7 months (Table 1).

### Prevalent mutation screening and sequencing results

High-frequency mutation screening and direct sequencing of *SLC25A13* gene in this infant revealed a paternally-inherited mutation c.851_854del4, as well as three heterozygous SNPs rs6957975 (IVS4+6G/A), rs6962870 (IVS10-84A/G) and c.1194A/G (exon 12). However, the maternal mutation was not detected. Since the primers for detecting the above mutation and SNPs were located in the flanking sequences of exons 4, 8, 9, 11 and 12, respectively, the possibility was ruled out for the maternally-inherited mutation to involve these 5 exons.

### Detection of citrin protein

**Figure 1:**
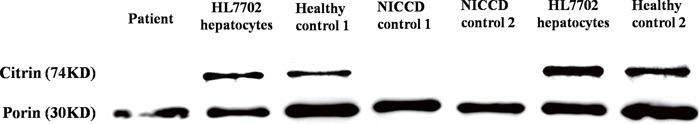
Western blotting analysis of mitochondrial proteins Citrin signal was detected in the cultured PBLs of two healthy controls and the HL7702 hepatocyte line, but absent in the cultured PBLs of this patient, a patient control homozygous for c.851_854del4 mutation (NICCD control 1), and another patient control heterozygous for mutations c.851_854del4 and [c.2T>C; c.1452+1G>A] (NICCD control 2).

As shown in Figure 1 and Supplementary Figure 1, the normal immunoreactive bands (74kD) against anti-citrin antibody were both detected in the cultured PBLs from all the healthy controls and a human hepatocyte line HL7702. Meanwhile, the citrin signal in HL7702 hepatocytes was relatively higher than that in PBLs, indicating that citrin were expressed just at a lower level in PBLs than in hepatocytes. Of particular note, citrin signal could not be detected in the cultured PBLs from this patient and the NICCD controls (Figure 1). These results provided reliable molecular evidences, at protein level, for the definite diagnosis of NICCD in the present infant.

### Results of cDNA cloning analysis

Based on the above findings, *SLC25A13* cDNA cloning using PBLs was then performed to explore molecular clues, at mRNA level, that might facilitate the identification of the hidden maternal *SLC25A13* mutation. As shown in Table 2, a total of 24 transcripts were detected, and all of which were transcribed from the paternally-inherited *SLC25A13* allele harboring the mutation *r*.851_854del4. These findings strongly suggested that the maternal mutation might be a large insertion/deletion resulting in an abnormal size of transcriptional product, or a mutation with involvement of the promoter region, which silenced the expression of the maternal *SLC25A13* allele.

**Table 2:** The *SLC25A13* ASVs detected by cDNA analysis using PBLs of the NICCD patient

Alleles	ASVs	Annotations	Clones
Paternally-inherited	*r*.851_854del	Transcripts with *r*.851_854del4	4
	*r*.213_328del;*r*.851_854del	Transcripts with *r*.851_854del4 and exon 4 skipping	3
	*r*.755_848;*r*.851_854del	Transcripts with *r*.851_854del4 and exon 8 skipping	4
	*r*.851_854del;*r*.1230_1231ins24	Transcripts with *r*.851_854del4 and *r*.1230_1231ins24	1
	*r*.213_328del;*r*.851_854del;*r*.1311_1312ins75	Transcripts with *r*.851_854del4, exon 4 skipping, and *r*.1311_1312ins75	2
	*r*.851_854del (Just sequencing the exons 8 and 9)	Transcripts with *r*.851_854del4	10
Maternally-inherited	No Transcripts detected		

The ASVs in this table were described according to the website http://www.hgvs.org/mutnomen/; Nucleotide numbering was based on cDNA sequence (GenBank:NM 014251), with +1 indicating the A of the ATG-translation initiation codon.

### Findings of semi-quantitative PCR analysis and family SNP analysis

**Figure 2:**
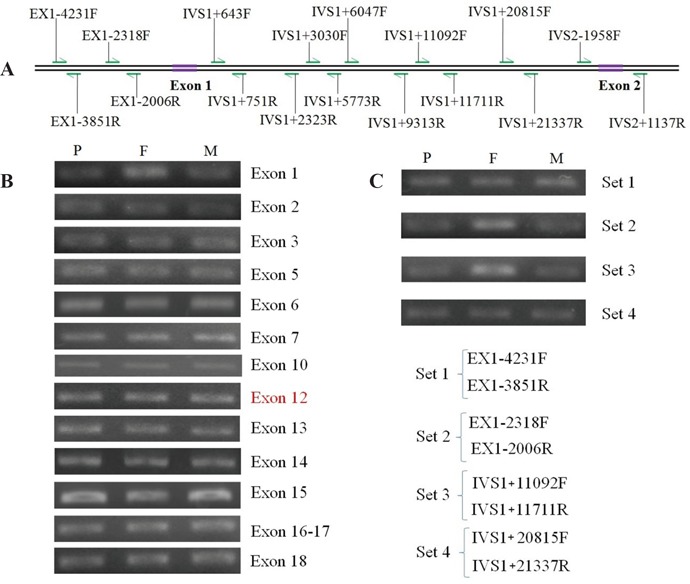
Results of semi-quantitative PCR and primers in positioning analysis of the novel large deletion **(A)** The relative positions of primers. **(B)** The semi-quantitative PCR products of *SLC25A13* exons with exon 12 (in red) as the internal control. Note that the product intensity in the patient (P) and mother (M) was both weaker than that in father (F) in the lane of exon 1, which was not the case in other exon lanes. **(C)** The semi-quantitative PCR products using the primer sets 1 to 4. The PCR products with primes sets 2 and 3 were both reduced in the patient and his mother. Primer sets and their nucleotide sequences were shown in Table 3.

In order to precisely localize the maternally-inherited mutation, semi-quantitative PCR amplification of the rest 14 exons other than the exons 4, 8, 9 and 11 were performed. When compared with the PCR product of exon 12, which was set as the internal control, similar signal intensities were obtained for all the remaining 12 exons but exon 1 (Figure 2B and Supplementary Table 1). The reduced amount of amplicons for exon 1 in the patient (P) and his mother (M), but not father (F), clearly indicated that the maternally-inherited mutation might locate around exon 1.

In turn, five primer sets A to E (Figure 2A and Table 3) were designed to analyze the SNPs around exon 1 in the family. We genotyped 7 SNPs within the DNA fragment from IVS1+3106 to IVS1+16630 (Table 4), and their family distribution could not be explained by way of Mendel's law of inheritance. For instance, the genotype at IVS1+3106 was C/G in the father and G/G in the mother, but it came out to be C/C in the infant. The SNP analytic results further indicated the existence of a maternally-originated large insertion/deletion around exon 1 in the patient.

**Table 3:** Primer sets for semi-quantitative PCR and familial SNP analysis

Sets	Primers	Sequences from 5′ to 3′ end	Products (bp)
A	Ex1-2318F	AAGTCTTCTGGGCTTTGTTGAAC	3113
	IVS1+643R	AATGCTCCGAGGCGGGTATCT	
B	IVS1+751F	CCAGAGGAAAGGCAACTCCAACAC	1573
	IVS1+2323R	TCATTTCCTCTTTGCTTTGGCTGG	
C	IVS1+3030F	CCTTTGGGCATCACCTTTATCGG	2744
	IVS1+5773R	GCAGGGATTTGCGATCTGGTTG	
D	IVS1+6047F	TCTTCCACATTGTTCACCCATCAT	3267
	IVS1+9313R	GACAAACAGTCGTCGTCACTTATGC	
E	IVS1-1958F	GTTCGTCCTACCCTACCTTTCTCAG	3149
	IVS2+1137R	ATTTGACACCCTGGTCCTCTTTCC	
1	Ex1-4231F	GTAAACTGAATGATAGGCCCCCTAAAT	405
	Ex1-3851R	ATTATGAAGGCCAGATGTCCGAAGT	
2	Ex1-2318F	The same as in set A	313
	Ex1-2006R	CTACCAGGATGTCCAGACGAGAC	
3	IVS1+11092F	GCATTTCTGCCTTCTACTGAGCC	467
	IVS1+11711R	AGTTGAGGGTCACAGAGGCAGTC	
4	IVS1+20815F	GTGGCTACATTTCTGTGGCTTCC	523
	IVS1+21337R	GGGTCTTCAAACACACTCACCAAAC	

The relative position of each primer is shown in Figure 2.

**Table 4:** *SLC25A13* SNPs in the family on Sanger sequencing

No.	SNPs	Position	Genotypes		
			Patient	Father	Mother
1	rs11975883	IVS1+3106	C/C	C/G	G/G
2	rs11982522	IVS1+3338	C/C	C/T	T/T
3	rs11975076	IVS1+3507	A/A	A/G	G/G
4	rs13237399	IVS1+3770	C/C	C/T	T/T
5	rs4551263	IVS1+3798	C/C	C/T	T/T
6	rs11773446	IVS1+6769	C/C	C/T	T/T
7	rs34257126	IVS1+16630	G/G	G/T	T/T

For the purpose of exploring the border of the hidden mutation, further semi-quantitative PCR approaches were carried out using the primers sets 1 to 4 (Figure 2 and Table 3). As a result, the PCR products with primes sets 2 and 3 were both reduced in the patient and his mother, indicating that the mutation might locate between the primers EX1-4231F (forward primer of Set 1) and IVS1+21337R (reverse primer of Set 4).

### Identification of the hidden mutation

**Figure 3:**
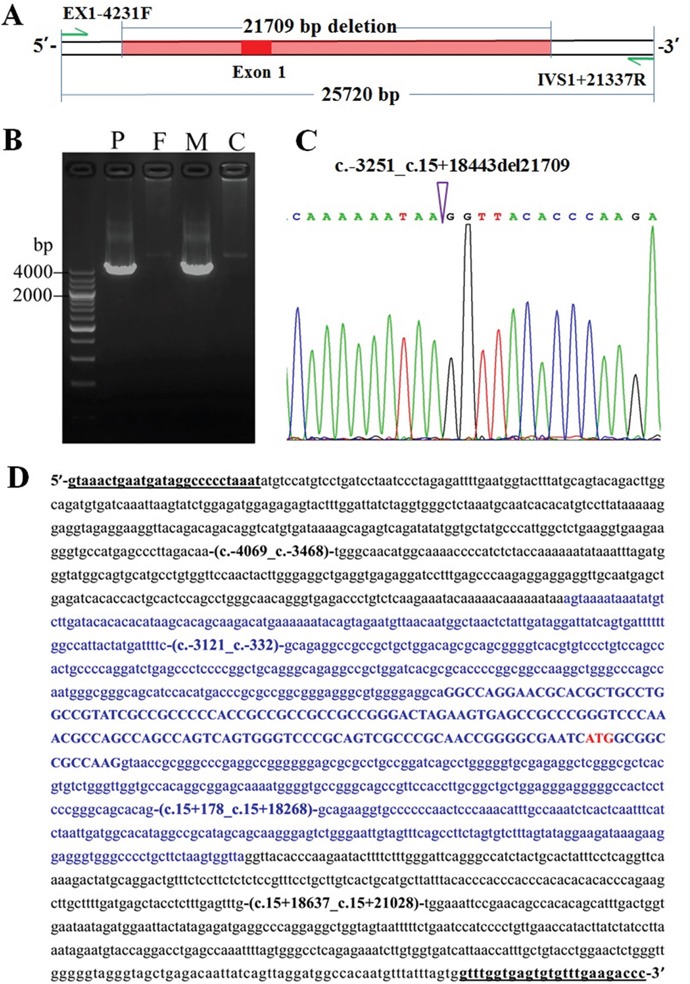
The large *SLC25A13* deletion identified in the infant and his mother **(A)** The positions of the primers EX1-4231F and IVS1+21337R and the novel large deletion. **(B)** LA-PCR with the primers EX1-4231F and IVS1+21337R yielded an unexpected 4000 bp band in the patient (P) and his mother (M), but not in the father (F) and normal control (C). **(C)** Sanger sequencing result of the unexpected PCR product, with the arrowhead indicating the breaking point. **(D)** The large deletion (all in blue) encompassed the entire exon 1 (blue upper-case, 152bp, accession number: AF118838.1), its downstream 18443bp within intron 1 (blue lower-case), as well as its upstream 3114bp (blue lower-case) where locates the promoter region. The initiation codon was labeled in red, with +1 indicating the nucleotide A in this codon. Underlined were the nucleotide positions of the primers EX1-4231F and IVS1+21337R, respectively.

Based on the findings above, another LA-PCR approach was conducted using the primers EX1-4231F and IVS1+21337R (Figure 3A), and an unexpected product of about 4000 bp in size in the patient and his mother, but not in the father, was detected (Figure 3B). The expected product was 25720 bp in size, which were too large to be amplified in this approach. The subsequent Sanger sequencing of the unexpected product uncovered a large deletion c.-3251_c.15+18443del (Figure 3C), which spans a DNA fragment of 21709bp in size, covering the promoter region and the entire exon 1 in the gene *SLC25A13* (Figure 3D).

Analysis of the deletion breakpoint-surrounding sequences using Repeatmaker (http://www.repeatmasker.org/cgi-bin/WEBRepeatMasker/) revealed an AluJr element adjacent to the upstream of the proximal breakpoint, an LTR/ERVL element located 21 bases upstream of the distal breakpoint, and a 1bp microhomology at the breakpoint junction (Supplementary Figure 2).

## DISCUSSION

Citrin protein (AGC2) is well-known to be expressed in the liver, kidney, and skin fibroblasts [1, 4, 27, 29, 44–46]. As a more accessible sample source, PBLs had been used to analyze *SLC25A13* mRNA by cDNA cloning [47] and to detect citrin protein by Western blotting [43]. However, although not infeasible, the Western blotting detection of citrin protein using fresh PBLs was a little bit difficult to repeat. A possible explanation might be the rather limited amount of mitochondria arising from the rather limited cytoplasm in contrast with the large nucleus size in PBLs, which leads to the relative trace expression of citrin protein in such cells [48]; meanwhile, the large quantities of nonspecific proteins relative to trace citrin signal might be another contributing interference factor. To address these issues, the PBLs in this study were expanded by cell culturing and then the mitochondria were isolated by differential centrifugation, following which the mitochondrial proteins were extracted for Western blotting analysis, thus eliminating the interference from nonspecific proteins in the cytoplasm and nuclei of the PBLs. As shown in Figure 1, by using this approach, citrin protein was well detected in the healthy control and hepatocyte line, but absent in this patient and the NICCD control. Our Western blotting method demonstrated satisfactory repeatability, and might be a new minimally-invasive and more-feasible molecular tool for NICCD diagnosis.

In fact, the mononuclear cells isolated from fresh blood by using Ficoll density gradient centrifugation, as in this study, consist of various cell types, including T (~70%), B (~15%), and natural killer (NK) (~10%) lymphocytes as well as a small amount of monocytes (~5%) [49, 50]. In this paper, we used mitogen/cytokine cocktail composed of phytohemagglutinin (PHA) and interleukin-2 (IL-2) to stimulate T, B and NK cells expansion. As a lectin, PHA has a high affinity for T lymphocyte surface receptors with mitogenic properties [51, 52], but actually, both T and B cells underwent mitosis in PHA-stimulated lymphocyte cultures, and the frequency of the mitotic B cells varied between 6-20% [53, 54]. Meanwhile, IL-2 mediates cell proliferation via its receptors which are primarily expressed on T and NK cells [55, 56]. We observed that the majority of the monocytes in this study were dying out in the first week of cell culture, which was in agreement with the previous findings by other group [57]. Overall, by way of PHA and IL-2 stimulation, we harvested enough amounts of expanded PBLs for the extraction of mitochondria proteins, which demonstrated satisfactory citrin signal on subsequent Western blotting analysis.

In this study, the prolonged jaundice, cholestatic biochemical alterations, metabolome findings as well as the benign prognosis in response to lactose-free and MCT-enriched formula all suggested NICCD diagnosis in the patient. The conventional DNA analytic methods only unraveled a paternally-originated *SLC25A13* mutation c.851_854del4. However, the results of Western blotting in Figure 1, of particular note, clearly indicated a NICCD diagnosis for the present patient, providing a reliable basis for the subsequent in-depth exploration of the maternal mutation. It was on this solid foundation that, by using semi-quantitative PCR and family SNP analysis as well, the hidden maternally-inherited *SLC25A13* mutation was precisely positioned and finally identified as a large deletion c.-3251_c.15+18443del21709bp. The in-depth molecular dissection of this NICCD case proved the significance of citrin protein detection by way of the Western blotting approach using mitochondria proteins extracted from expanded PBLs, and further supported the concept that *SLC25A13* cDNA cloning analysis using PBLs along with semi-quantitative PCR analysis and SNP analysis were effective tools to facilitate the identification of large *SLC25A13* deletion/insertion mutations.

To the best of our knowledge, the novel c.-3251_c.15+18443del21709bp mutation is the hitherto largest deletion in the *SLC25A13* mutation spectrum. This 21.7kb deletion spanned from 3251 bp upstream of the initiation codon to 18443bp downstream of the exon 1, covering the promoter region and the entire exon 1 where the initiation codon was located, hence completely silencing the transcriptional and translational expression of the affected *SLC25A13* allele. This gross deletion, as illustrated in Supplementary Figure 2, might be resulted from a non-homologous end joining (C-NHEJ) mechanism, which was involved in repairing a diverse range of substrate DNA ends at double-strand break (DSB), following by end joining in the absence of extensive sequence homology and with very short stretches of sequence identity (a few base pairs) between the two ends of the breakpoint junctions [58, 59]. The paternally-originated mutation c.851_854del4 theoretically leads to a frameshift from position 284 and introduced a stop codon at position 286, leading to the production of a truncated protein [1], which might be unstable and soon degraded, and hence could not be detected on Western blotting analysis [42–44]. Consequently, cDNA cloning analysis using PBLs from the patient just uncovered alternative splicing variants (ASVs) with *r*.851_854del from the paternally-inherited *SLC25A13* allele, as shown in Table 2, but no citrin protein could be detected in the patient on Western blotting analysis, as shown in Figure 1.

In conclusion, via sophisticated molecular analysis using PBLs, this infant was definitely diagnosed to have NICCD with the *SLC25A13* genotype of c.851_854del/c.-3251_c.15+18443del21709. The identification of the novel gross deletion further supported that cDNA cloning analysis using PBLs, along with SNP analysis and semi-quantitative PCR, could facilitate the identification of novel large *SLC25A13* mutations. Of particular note, the Western blotting approach using mitochondrial protein extracted from expanded PBLs might be a new minimally-invasive and more-feasible molecular tool for NICCD diagnosis.

## MATERIALS AND METHODS

### Subjects and ethics

A cholestatic infant strongly suspected to have NICCD, his parents, two NICCD control homologous for mutation c.851_854del4 (for Western blotting), four healthy volunteers (one for LA-PCR analysis and all for Western blotting) and a human hepatocyte line HL7702 (Cell Bank of Chinese Academy of Science, China) were recruited as the research subjects in this study. Clinical findings were described as a case report. Written informed consents had been obtained from all healthy volunteers and parents of the patients prior to this research, which had been approved by the Committee for Medical Ethics, the First Affiliated Hospital, Jinan University.

### Conventional DNA analysis

Genomic DNA was extracted from peripheral venous blood samples of the research subjects. The four high-frequency *SLC25A13* mutations c.851_854del4, c.1638_1660dup, c.615+5G>A and IVS16ins3kb were screened by means of PCR/LA-PCR and PCR-RFLP. If initial screening only revealed one or none mutation, all *SLC25A13* 18 exons and their flanking sequences were amplified by PCR/LA-PCR, and then Sanger sequencing was carried out to explore the obscure *SLC25A13* mutation(s). Following that, if either of the biallelic pathogenic mutations still remains unclear, the NICCD possibility will be evaluated at protein level using a Western blotting approach to detect the citrin protein in mitochondria proteins of cultured PBLs.

### Cell culture

H7702 hepatocytes were grown as monolayer in the complete medium [RPMI medium 1640 (L-glutamine+, Gibco, USA), 10% Fetal Bovine Serum (FBS, Gibco, Australia) and 1% Penicillin/Streptomycin (Gibco, USA)] at 37°C in a humidified 5% CO_2_ atmosphere [60, 61]. PBLs were isolated from 1ml of fresh EDTA-anticoagulant peripheral venous blood with lymphocyte separation medium (LSM, Ficoll-hypaque, density at 20°C: 1.077-1.080g/ml; MP Biomedicals, USA) and washed twice with 1× phosphate buffer saline (PBS). The PBLs were then adjusted to a density of 3-5 ×10^5^cells/ml by diluting with the above complete medium in the presence of 5μg/ml of PHA (Sigma, USA) and 10 ng/ml of recombinant IL-2 (Peprotech, USA) [54, 62, 63]. For subcultures, the high-density cultures of PBLs were diluted with the above stimulative medium down to an appropriate seeding density every 2-3 days.

### Mitochondrial protein extraction

Mitochondria were isolated by mechanical means and differential centrifugation [64] in accordance to the manufacturer's instructions in a mitochondrial protein extraction kit (Bestbio, China). Briefly, a total of 2 × 10^7^ cells were harvested by centrifugation at 500×g for 5 min and washed 3 times with 1×PBS. The cells were resuspended with Solution A and homogenized on ice with a glass homogenizer. Following that, a low-speed spin (1,000×g) at 4°C for 5 min pelleted unbroken cells, membranes, and nuclei. The supernatant underwent a subsequent high-speed centrifugation (15,000×g, 20 min) to sediment mitochondria, which were then resuspended in Solution B and subjected to the same high-speed spin. Protein was extracted from the crude mitochondria, which were disrupted in protein lysis buffer with protease inhibitors cocktail [4-(2-aminoethyl) benzenesulfonyl fluoride, aprotinin, E-64, leupeptin, bestatin, EDTA and pepstatin A], and kept at −80°C until used.

### Western blotting analysis

A total of 20 μg of protein were loaded onto a 10% sodium dodecyl sulfate (SDS)-polyacrylamide gel and then transferred to a polyvinylidene fluoride (PVDF) membrane. Citrin protein was detected by using anti-citrin IgG (N-terminal region, 1-285 codons; diluted 1: 3,000) [4, 43, 44] as a first antibody with horseradish peroxidase conjugated goat anti-rabbit IgG as a second antibody (diluted 1: 6,000, Boster, China). Meanwhile, porin protein, as a loading control, was probed with an anti-porin antibody (diluted 1: 3,000; Sangon Biotech, China) [24]. Immunoreactive bands were visualized with ECL substrate (Bio-Rad, USA) in a chemiluminescence imaging system (Kodak Gel Logic 1500, USA).

### *SLC25A13* cDNA cloning analysis

According to the Western blotting result, RT-PCR and cDNA cloning were performed, as in our previous publications [11, 46], to explore aberrant mRNA molecules as clues facilitating the identification of the hidden *SLC25A13* mutation. Briefly, PBLs were isolated from heparinized blood samples of patient, total RNA was extracted using Trizol (Invitrogen, USA), and then RNA was reversely transcribed to cDNA immediately by means of MMLV reverse transcriptase (Promega, USA). Following the nested PCR procedure, the amplified *SLC25A13* ORF products were purified and cloned into pMDTM18-T Vector (Takara, China). The recombinant vectors were then transformed into DH5α *E. coli* competent cells. After cultured for 12-16 hours at 37°C, positive clones were subsequently selected and sequenced.

### Positioning and identification of the large deletion

Based on the above findings, semi-quantitative PCR analysis and familial SNP analysis were conducted to precisely position the hidden mutation in this case [35]. With the primers as described in the literature [65], the semi-quantitative PCR was carried out on 14 *SLC25A13* exons other than the exons 4, 8, 9 and 11, with the exon 12 as the internal control. With the same amount of genomic DNA as amplified templates, a 26–28 cycles of amplification were performed to ensure that the amplification stopped at the exponential growth phase. We used the quantitative software to quantify the density of electrophoresis bands by converting them into digital form and compared the radio of each exon gray level among the patient and his parents. The mutation was then positioned around the positive exon, which yielded the reduced amount of amplification products in the patient and his mother.

Meanwhile, PCR products, with genomic DNA as template and by using the primer Sets A to E (Table 3 and Figure 2) which all located around the above positive exon, were sequenced to analyze the SNPs in all family members. And then, according to the findings of familial SNP analysis, new semi-quantitative PCR were performed using the primers Sets 1 to 4 (Table 3 and Figure 2) to explore the border of the mutation.

Finally, another LA-PCR was performed using primers Ex1-4231F and IVS1+21337R (Table 3 and Figure 2), with an expected product of 25720bp in size. The PCR temperature profile was 94°C for 5 min followed by 35 cycles at 98°C for 12s, 60°C for 40s, 68°C for 8 min, and a final extension step at 72°C for 10min. The unexpected PCR products were then sequenced to uncover the maternal mutation.

## SUPPLEMENTARY FIGURES AND TABLE


