# Supplementary material for: Molecular diagnosis of citrin deficiency in an infant with intrahepatic cholestasis: identification of a 21.7kb gross deletion that completely silences the transcriptional and translational expression of the affected SLC25A13 allele
Source: Oncotarget. 2017 Aug 3;8(50):87182–93. doi: 10.18632/oncotarget.19901 (PMC5675625; doi:10.18632/oncotarget.19901)
Supplement: Supplementary file 1 [file oncotarget-08-87182-s001.pdf]

Molecular diagnosis of citrin deficiency in an infant with intrahepatic cholestasis: identification of a 21.7kb gross deletion that completely silences the transcriptional and translational expression of the affected *SLC25A13* allele

SUPPLEMENTARY MATERIALS

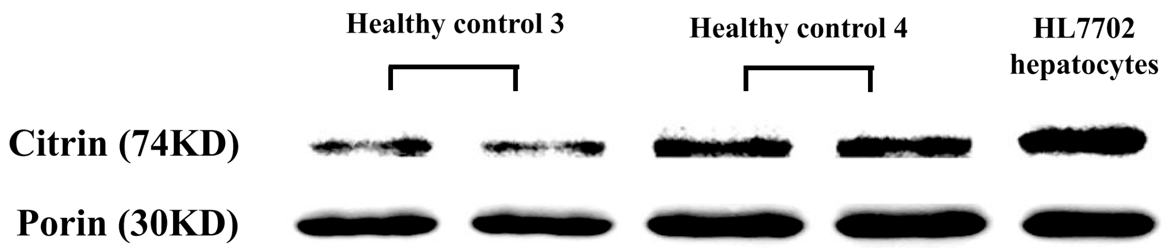

Supplementary Figure 1: Additional Western blotting analysis of mitochondrial proteins. Citrin signal was detected in the cultured PBLs of two healthy controls and the HL7702 hepatocyte line.

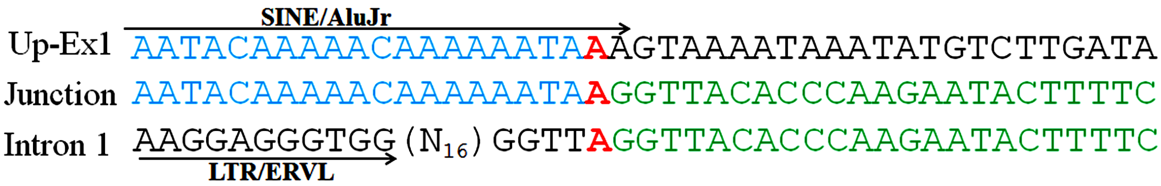

Supplementary Figure 2: Nucleotide sequences around the deletion breakpoints and the deletion junction. The Upper (Up-Ex1) and lower (Intron 1) lanes in this figure represented the normal reference sequences around the proximal and distal deletion breakpoints, respectively. The nucleotides in black marked partial deleted sequences. The middle lane (Junction) indicated the patient's DNA sequence around the deletion junction. The nucleotide A in bold red indicated a microhomology base. The nucleotides in blue represented the upstream sequences of the proximal breakpoint while those in green, the downstream of the distal breakpoint. The elements of SINE/AluJr and LTR/ERVL were both underlined by arrows. LTR, long terminal repeat; ERVL, endogenous retrovirus; SINE, short interspersed nuclear element; AluJr, subfamily of Alu element.

Supplementary Table 1: Signal intensity of the electrophoresis bands for the PCR products in Figure 2

PCR products	Signal intensity			Relative intensity*		
	Patient	Father	Mother	Patient	Father	Mother
Exon 1	62.76	92.51	73.46	0.7	1.0	0.7
Exon 2	77.85	72.34	70.48	0.9	0.8	0.7
Exon 3	77.03	82.16	92.1	0.8	0.9	0.9
Exon 5	99.13	95.75	97.27	1.1	1.0	1.0
Exon 6	98.69	93.63	100.31	1.1	1.0	1.0
Exon 7	95.66	106.71	118.24	1.1	1.1	1.2
Exon 10	102.34	106.76	109.44	1.1	1.1	1.1
Exon 12	90.66	93.07	95.63	1.0	1.0	1.0
Exon 13	107.06	100.54	98.87	1.2	1.1	1.0
Exon 14	89.98	84.73	88.96	1.0	0.9	0.9
Exon 15	118.2	101.86	119.08	1.3	1.1	1.2
Exon 16-17	95.42	103.06	110.47	1.1	1.1	1.1
Exon 18	83.33	92.15	99.8	0.9	1.0	1.0
**Primer sets around exon 1**						
Set 1	86.51	89.61	98.85	1.0	1.0	1.0
Set 2	74.78	97.64	77.3	0.8	1.0	0.8
Set 3	88.43	112.94	81.97	1.0	1.2	0.8
Set 4	77.72	76.27	78.32	0.9	0.8	0.8

* The signal intensity of every PCR bands relative to that of the internal control (Exon 12).
